# ATP-binding cassette systems in *Burkholderia pseudomallei *and *Burkholderia mallei*

**DOI:** 10.1186/1471-2164-8-83

**Published:** 2007-03-28

**Authors:** David N Harland, Elie Dassa, Richard W Titball, Katherine A Brown, Helen S Atkins

**Affiliations:** 1 Defence Science and Technology Laboratory, Porton Down, Salisbury, Wiltshire, SP4 0JQ, UK; 2 Départment de Microbiologie Fondamentale et Médicale, Institut Pasteur, Paris Cedex 15, France; 3 Department of Infectious and Tropical Diseases, London School of Hygiene and Tropical Medicine, London, WC1E 7HT, UK; 4 Division of Cell and Molecular Biology, CMMI, Flowers Building, Imperial College London, London SW7 2AZ, UK

## Abstract

**Background:**

ATP binding cassette (ABC) systems are responsible for the import and export of a wide variety of molecules across cell membranes and comprise one of largest protein superfamilies found in prokarya, eukarya and archea. ABC systems play important roles in bacterial lifestyle, virulence and survival. In this study, an inventory of the ABC systems of *Burkholderia pseudomallei *strain K96243 and *Burkholderia mallei *strain ATCC 23344 has been compiled using bioinformatic techniques.

**Results:**

The ABC systems in the genomes of *B. pseudomallei *and *B. mallei *have been reannotated and subsequently compared. Differences in the number and types of encoded ABC systems in belonging to these organisms have been identified. For example, ABC systems involved in iron acquisition appear to be correlated with differences in genome size and lifestyles between these two closely related organisms.

**Conclusion:**

The availability of complete inventories of the ABC systems in *B. pseudomallei *and *B. mallei *has enabled a more detailed comparison of the encoded proteins in this family. This has resulted in the identification of ABC systems which may play key roles in the different lifestyles and pathogenic properties of these two bacteria. This information has the potential to be exploited for improved clinical identification of these organisms as well as in the development of new vaccines and therapeutics targeted against the diseases caused by these organisms.

## Background

The *Burkholderia *are non spore-forming, Gram-negative bacteria. *Burkholderia pseudomallei *is a saprophytic bacillus and the causative agent of the emerging infection melioidosis, an endemic disease in southeast Asia and Northern Australia [1].*Burkholderia mallei *is an obligate pathogen of solipeds and can infect humans although cases are rare [2]. It is the causative agent of glanders, a disease that is endemic in areas of Asia, the Middle East, Northern Africa and the Mediterranean [3]. Both *B. mallei *and *B. pseudomallei *have been classified as category B threat agents by the US Center for Disease Control and Prevention.

The reporting of the sequenced genomes of both *B. pseudomallei *strain K96243 [4] and *B. mallei *strain ATCC 23344 [5] has revealed that these bacteria are closely related. Multilocus sequence-based typing has shown that *B. mallei *is a clonal derivative of *B. pseudomallei *[6], although significant differences exist between the genomes of these organisms [4,5]. The *B. pseudomallei *K96243 genome is 1.31 Mb larger than that of *B. mallei *ATCC 23344 and 16% of chromosome 1 and 31% of chromosome 2 of *B. pseudomallei *K96243 is unique with respect to *B. mallei *ATCC 23344. In contrast, < 1% of chromosome 1 and only 4% of chromosome 2 of *B. mallei *ATCC 23344 is unique with respect to *B. pseudomallei *K96243. The acquisition of DNA is a feature of the genome of *B. pseudomallei *K96243 [5], whilst the reduced size of the *B. mallei *ATCC 23344 genome appears to be related to a process of reductive evolution [4]. The difference in genome sizes may be related to differences in lifestyle observed between these two organisms. Specifically, the greater number of genes in *B. pseudomallei *may reflect the ability of the organism to survive in soil and water as well as in a diverse range of mammalian and avian hosts whereas *B. mallei *survives mainly within soliped hosts and not in the environment [3]. Furthermore, comparative genomic analyses indicate that *B. mallei *may lack gene products and pathways essential for survival in the environment whereas both organisms share other functional pathways associated with virulence or drug resistance [5,4].

ATP-binding cassette (ABC) systems are versatile transport systems which vary greatly in size and generally function to import or export a range of substances [7]. In pathogenic bacteria these systems can play important roles in survival and pathogenicity [7]. The common feature of all ABC systems is the hydrolysis of ATP to ADP by the highly conserved ABC [8]. The ABC has three conserved motifs, known as the Walker A and B sites, commonly found in ATPases, and the linker peptide which has the signature motif LSSGQ. The majority of ABC systems contain two hydrophilic cytoplasmic domains (ABC domains) in association with two hydrophobic membrane-spanning domains (IM domains). Import systems are specific to prokaryotic organisms and contain ABC and IM domains on separate polypeptide chains along with an extracytoplasmic binding protein (BP) for proper function. In Gram-negative bacteria the BPs are located in the periplasm whereas in Gram positive organisms, these proteins are anchored to the cellular membrane by an N-terminal acyl glyceryl cysteine. An important role for bacterial ABC import systems is in the import of iron-siderophore complexes, free iron and other iron bound complexes. Bacterial ABC import systems function in the acquisition of free iron, iron-bound complexes, mono-and oligosaccharides, organic and inorganic ions, amino acids and short peptidesmetals and vitamins [9]. In contrast, ABC systems involved in export are found in both prokaryotic and eukaryotic organisms contain IM and ABC domains fused in a variety of conformations. Most export systems are expressed as with the IM domain fused to the ABC either N-terminally (IM-ABC) or C-terminally (ABC-IM). These polypeptides homodimerise to form a functional system. A great number are predicted to have a role in drug resistance [9], although a number have other functions such as in the secretion of siderophores [10]. Other ABC systems with roles regulatory functions such as DNA repair [9] do not contain IM domains and often are comprised of two fused ABC domains (ABC2). ABC systems are also involved in toxin export and include the well-characterized system for haemolysin export in *Escherichia coli *[11,12]. Recently, ABC system components have been identified as potential vaccine targets due their cellular location in or near bacterial membrane and their potential presentation to the host immune system [13].

The aim of this study was to carry out a detailed comparison of the ABC systems of the genomic sequencing strains *B. pseudomallei *strain K96243 and *B. mallei *strain ATCC 23344. Reconstruction of the annotation of the ABC systems using uniform classification was undertaken in order to give a precise prediction of ABC system complements. Data obtained has the potential to provide new insights into the types of proteins used for the survival and pathogenesis of these two related but functionally distinct organisms.

## Results and Discussion

The original annotations of ABC systems in the genomes of *B. pseudomallei *strain K96243 and *B. mallei *strain ATCC 23344 are somewhat imprecise since a uniform nomenclature for these proteins was not used and functional assignment varied considerably. Here we describe the results of the reannotation and a comparison of the ABC systems of both organisms. New information about the predicted function of ABC systems along with similarities and differences between the organisms has been achieved through the use of the ABCISSE database. This approach has enabled an improvement in the identification and assignment of components of specific ABC systems with uniform annotation and classification. A significant advantage of this work, compared to the existing annotations, is the prediction of how the individual ABC system components combine to form functional systems. In particular, ABC system-associated proteins are included which are not encoded within short operons, but rather are shown to be part of a system on the basis of similarity to proteins with known or predicted function in a specific system type. A complete inventory of the ABC systems of *B. pseudomallei *strain K96243 and *B. mallei *strain ATCC 23344 is included as an additional file.

### The ABC systems of *B. pseudomallei*

**Figure 1:**
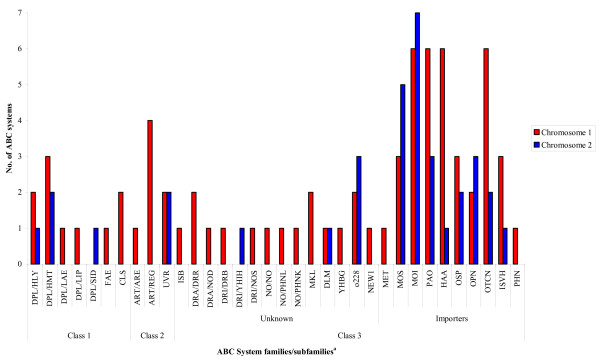
Chromosomal distribution of ABC systems in *B. pseudomallei *K96243. ^a ^Families and subfamilies as described at the ABSCISSE database [34].

An analysis of the 7.2 Mb genome of *B. pseudomallei *strain K96243 revealed a total of 338 ABC system-associated open reading frames (ORFs) organised into 105 predicted functional ABC systems. Of these, 70 systems (66.6%) are encoded on chromosome 1 and 35 systems (33.3 %) are encoded on chromosome 2 (Figure 1). Chromosome 1 of *B. pseudomallei *K96243 represents 56% of the genome and contains a higher proportion of coding sequences (CDSs) than chromosome 2. Chromosome 1 also contains a higher proportion of CDSs predicted to be involved in core metabolic functions, whilst chromosome 2 preferentially encodes proteins with accessory functions [5]. Based upon the analysis undertaken here chromosome 1 of *B. pseudomallei *K96243 is predicted to encode 25 systems that belong to 19 different families (and subfamilies) which are not encoded on chromosome 2. In contrast, chromosome 2 encodes only two predicted systems that do not belong to families already encoded on chromosome 1.

The ABC systems of *B. pseudomallei *K96243 were classified according to the format described by Dassa and Bouige [9] including three main classes. Fourteen systems were ascribed to class 1 (Figure 1) which is characterised by an ABC domain fused to an IM domain and contains systems predicted to function in the export of substances from cells. Nine systems with a duplicated, fused ABC were found to belong to class 2, with predicted functions in antibiotic resistance via an undetermined mechanism or intracellular regulatory processes. However, the majority of ABC systems of *B. pseudomallei *K96243 (82 systems) were found to belong to class 3, predicted to function in import processes. These class 3 ABC systems could then be further subdivided. Sixty-one systems were predicted to be binding protein-dependent importers of known function and eleven systems were predicted to be importers but of unknown function. The remaining ten systems, although clustered phylogenetically in class 3, failed to show signature sequences indicative of known import functions.

The large number of systems encoded on the *B. pseudomallei *strain K96243 genome perhaps reflects the diverse environment in which the organism can survive [14]. The acquisition of genomic material, including additional ABC systems, during the evolution of *B. pseudomallei *may have enhanced the capacity of this organism to survive in different environments and in a range of mammalian hosts [4]. With the exception of one putative monosaccharide import system, disrupted by an apparent deletion of the ABC, the genome of *B. pseudomallei *strain K96243 was predicted to encode all of the systems present in *B. mallei *strain ATCC 23344 (see additional file 1). In addition, *B. pseudomallei *strain K96243 was found to have a further 29 predicted systems which are either absent or predicted to be inactive in *B. mallei *strain ATCC 23344 (see additional file 1). The analysis also indicated that some ABC systems may share the same function. This higher level of redundancy and larger number of ABC systems in *B. pseudomallei *compared to *B. mallei *ATCC 23344 may contribute to the ability of *B. pseudomallei *may contribute to survive in a range of environments and hosts.

### The ABC systems of *B. mallei*

**Figure 2:**
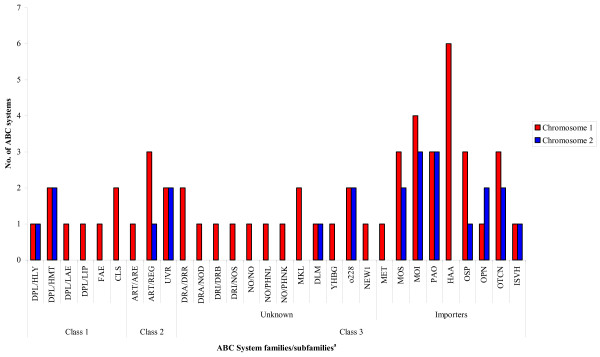
Chromosomal distribution of ABC systems in *B. mallei *ATCC 23344.

*B. mallei *strain ATCC 23344 has a genome size of 5.8 Mb and is predicted to encode a total of 275 ABC component proteins forming 77 different ABC systems. Of these, reannotation identified 54 systems (70%) encoded on chromosome 1 and 23 systems (30%) encoded on chromosome 2 (Figure 2). The class distribution of ABC systems in *B. mallei *ATCC 23344 was found to be similar to that observed in *B. pseudomallei *K96243 described above. Specfically, 11 ABC systems of *B. mallei *ATCC 23344 are predicted to belong to class 1, 9 systems belong to class 2 and have ABC 2 arrangement, and a total of 57 systems are predicted to belong to class 3. The distribution of class 3 systems was also similar to that identified in *B. pseudomallei *strain K96243 with 39 predicted import systems of known function, 10 predicted import systems of unknown function, and 8 systems classified phylogenetically as class 3 but without a predicted functional mechanism.

*B. mallei *strain ATCC 23344 was found to have lost some or all of the apparent redundancy observed in *B. pseudomallei *K96243, especially for the uptake of polyamines, monosaccharides and polar amino acids, demonstrated by a reduced number of *B. mallei *ABC systems in the class 3 MOI, MOS and PAO families compared to *B. pseudomallei *(Figures 1 and 2). As stated above, *B. pseudomallei *strain K96243 encodes 27 more ABC systems than to *B. mallei *strain ATCC 23344 (see Table 1). Three of these ABC systems are located in the genome islands specific to *B. pseudomallei *strain K96243 [5] which suggest that they have been acquired since this organism diverged from *B. mallei*. Comparison of the reannotated ABC system inventories reveals that the *B. mallei *ATCC 23344 genome completely lacks 14 of the remaining 24 systems, whist another 7 systems are predicted to be inactive due to the deletion of a gene encoding one ABC component. Three other systems appear to be inactive due to the presence of a predicted pseudogene within the each system. Although some of the lost systems in *B. mallei *may be redundant due to the presence of other similar systems, the loss of other functional systems in *B. mallei *results in the complete absence of systems of particular families or sub-families. For example, *B*. *mallei *has no ABC systems for the uptake of pyochelin, polar amino acid, or alkylphosphonate (see additional file 1). Thus, it is possible that these allocrites may not be required by *B. mallei *in its reduced lifestyle compared to *B. pseudomallei*.

Reannotation and phylogenetic classification of the ABC systems of *B. mallei *strain ATCC 23344 has enabled the prediction of new functions involved in lipid A, haemolysin or ornibactin export, drug resistance, lipoprotein release and the import of iron (III), oligopeptides, or aliphatic sulphonates for ABC systems previously annotated without predicted functions. Genome reduction in *B. mallei *compared to *B. pseudomallei *appears to have resulted in the loss of metabolic properties essential for environmental survival [3]. In this context, the reduction in the number of ABC systems encoded by *B. mallei *described here may reflect the organism's ability to survive in its specialized environment within an animal host and concomitantly its inability to survive in the environment.

### ABC systems involved in virulence and drug resistance

Pore-forming toxins such as haemolysin have been previously identified as important virulence factors in a range of pathogens including *Escherichia coli, Pseudomonas aeruginosa *and *Corynebacterium diphtheriae *[15]. The genome of *B. pseudomallei *strain K96243 was found to encode three systems for the export of haemolysin from the cell as a result of the new reannotation and classification. The genome of *B. mallei *strain ATCC 23344 encodes orthologs of two of these systems. Interestingly neither *B. mallei *nor *B. pseudomallei *have demonstrated haemolytic activity and no haemolysins associated with these organisms have been characterized yet. Nevertheless, these results open the possibility that these *Burkholderia *strains may produce haemolysin-type macromolecules which, for example, could be expressed during the infection process within the host. Other macromolecules such as LPS and capsule polysaccharides have also been suggested to act as virulence factors in both *B. mallei *[16,17] and *B. pseudomallei *[18-20], offering protection against antibody- and complement-mediated immune responses. Orthologs of ABC systems predicted to be involved in the export of capsular polysaccharide and LPS have been identified through reannotation as present in *B. pseudomallei *K96243 and *B. mallei *ATCC 23344 (see additional file 1).

The reannotation described here has identified both *B. pseudomallei *and *B. mallei *ABC systems with predicted roles in resistance to MLS (macrolides, lincosamide and streptogramin), polyketide (such as rifamycin) and peptide antibiotics (such as colistins). It is known that *B. pseudomallei *is intrinsically resistant to most penicillins (although not β-lactams), first and second generation cephalosporins, macrolides, rifamycins, colistins and aminoglycosides [21]. Similarly, *B. mallei *is also resistant to these antibiotics, with the exception of aminoglycosides [22]. The mutants which disrupt function of the *B. pseudomallei *Resistance-Nodulation-Cell Division (RND) superfamily BpeAB-OprA [23] and Amr-OprA [24] efflux systems have both been shown to increase the susceptibility of this organism to aminoglycosides. *B. mallei *lacks an Amr-OprA system which likely contributes to its sensitivity to aminoglycosides Comparisons of the ABC system inventories here also indicate that a *B. pseudomallei *ABC system predicted to be involved in antibiotic resistance, but with no predicted specificity, is mutated in *B. mallei *strain ATCC 23344. This mutation may also contribute to the difference in antibiotic sensitivities observed between *B. mallei *and *B. pseudomallei *(see additional file 1).

### ABC systems involved in the iron acquisition

The reannotation and comparison of the ABC systems of *B. pseudomallei *strain K96243 and *B. mallei *strain ATCC 23344 reveals differences between the two organisms in the number of ABC systems potentially involved in the acquisition of iron.*B. pseudomallei *strain K96243 encodes eight predicted ABC iron acquisition systems whereas only four of these systems are present in *B. mallei *ATCC strain 23344 (Table 2). Two of these orthologous systems are predicted to be involved in the direct uptake of iron (III) from the environment, and the two other systems are predicted to be involved in iron acquisition using siderophores. About 15 years ago *B. pseudomallei *was shown to produce a siderophore, under iron-limiting growth conditions, which was named malleobactin [25]. It was subsequently shown that malleobactin acquires iron from transferrin and lactoferrin [26]. Recently, malleobactin was reisolated and shown, by mass spectrometry, to actually be composed of three compounds [27]. Though no chemical structures of these compounds have been determined yet, two of these compounds show characteristics similar to the ornibactins from *Burkholderia cenocepaci*a [27,28]. Specifically, both *B. pseudomallei *K96243 and *B. mallei *ATCC 23344 have an ABC system annotated for export of ornibactin, and another system associated with the import of ornibactin (although annotated for the import of hemin, this system is predicted to interact with one of a number of siderophore receptors including the ornibactin receptor). A recent comparative microarray study revealed that under iron-limited growth conditions ABC systems predicted to be involved in the export of ornibactin and in the uptake of iron-ornibactin complexes are up regulated in both *B. pseudomallei *K96243 and *B. mallei *ATCC 23344 [29]. However, four ABC iron acquisition systems present in *B. pseudomallei *strain K96243 do not appear to be functional in *B. mallei *strain ATCC 23344. Two of these systems, predicted to function in the direct uptake of iron (III) and hemin from the environment, are disrupted by pseudogenes in *B. mallei *ATCC 23344. In addition, the import of iron bound to the siderophore pyochelin is predicted to be non-functional as a result of an apparent deletion of the pyochelin outer membrane receptor. Finally, an additional ABC system involved in the export of pyochelin to the extracellular environment is completely absent from the genome of *B. mallei *ATCC strain 23344. Notably, a global transcriptional analysis of RNA extracted from *B. pseudomallei *under iron-limiting conditions shows upregulation of a pyochelin receptor, as well upregulation of a siderophore receptor designated for the import of malleobactin [27] (see also Table 2). Furthermore, comparative microarray data also shows that in iron-limited growth conditions, pyochelin export and import systems are upregulated in *B. pseudomallei *K96243 but not in *B. mallei *[29]. The ability to scavenge iron is particularly important for the survival of intracellular pathogens due to the low levels available in the host cell environment. For example, *Mycobacterium tuberculosis *has been demonstrated primarily to survive within host macrophages and iron uptake through siderophores is required for growth and virulence in the macrophage [30]. Iron-dependent regulation of virulence has also been described for *Pseudomonas aeruginosa*, a close relative of *B. pseudomallei*, and involves the expression of siderophores during iron-limited conditions which upregulate the expression of other virulence factors, including haemolysin [31,32]. In addition, iron overload conditions such as β-thalassaemia may predispose patients to infection with *Salmonella enterica *serotype Typhimurium and other intracellular pathogens [30]. It is known that the ability of *B. pseudomallei *to successfully establish an infection may be dependent on its ability to scavenge iron [33]. Thus, the comparatively reduced ability of *B. mallei *to scavenge iron using its ABC systems may correlate with the different lifestyle of this organism and may impact upon its virulence properties. A detailed investigation of the iron acquisition capabilities of *B. mallei *ATCC 23344 and *B. pseudomallei *K96243 and the influence of the ABC systems on virulence would enhance the understanding on the differences between these two organisms.

## Conclusion

In this study the ABC systems of *B. pseudomallei *strain K96243 and *B. mallei *strain ATCC 23344 have been reannotated using the ABCISSE database in order to provide new information and a uniform annotation and classification of ABC systems in these organisms. The complete set of ABC systems in *B. mallei *and *B. pseudomallei *is now incorporated into the ABCISSE database [34]. The new annotation has enabled a more detailed comparison to be made of the numbers and types of ABC systems present in these pathogenic *Burkholderia *species. The greater number and redundancy of ABC systems in *B. pseudomallei*, including those systems associated with iron acquisition, may be indicative of ability of this organism to persist and replicate in a greater variety of environments when compared to *B. mallei*. It is also worth noting that genomes of these bacteria encode a large number of ABC systems when compared to the predicted numbers of ABC systems in other organisms [35].*B. mallei *is an obligate mammalian pathogen and as such is predicted to require a smaller number of ABC systems. *B. mallei *is a recent divergent from *B. pseudomallei *[6] and is undergoing a process of reductive evolution [5] resulting in a reduced genome, a reduced the number of ABC systems and limited ability to survive in a variety of niches compared to *B. pseudomallei*. Interestingly, this process of reductive evolution still leaves *B. mallei *with a considerably higher number of ABC systems (77 in total) compared to the average number of 45 for obligate extracellular bacteria [35].

The differences in ABC systems identified here from comparison the genomes of *B. pseudomallei *strain K96243 and *B. mallei *strain ATCC 23344 may also be exploited for the development of new diagnostic or clinical reagents specific for each organism. For example, the addition of primers targeted to the different ABC systems of each organism could add another layer of accuracy to the existing PCR-based tests which use type III secretion systems [36] or 23S rDNA [37] for identification. Furthermore, since ABC systems have also been identified as candidates for vaccine development [13], the selection of outer membrane ABC components from *B. pseudomallei *and *B. mallei *may represent good targets for the development of new vaccines designed to provide protection against melioidosis or glanders.

## Methods

ABC systems in *B. pseudomallei *K96243 or *B. mallei *ATCC 23344 were identified on the basis of sequence homologies to proteins of ABC systems in other bacterial species. Proteins were classified according to their predicted roles as ABC system components. The complete CDSs (ORFs likely to encode proteins) of *B. pseudomallei *K96243[5]or *B. mallei *ATCC 23344[4] were used to query the ABCISSE v3.2 database [34] which includes ABC protein classification and predicted function based on similarity to proteins with experimentally derived function. The query was performed by using BLASTP [38], with a threshold e-value of 10^-6^. A hit from this search was predicted to belong to a given family (or subfamily) and to transport a specific allocrite if the top ten hits belonged to the family (or subfamily) and were predicted to transport a specific allocrite. An ABC system was defined as a series of contiguous ORFs that shared the same family, subfamily and substrate. Some additional ORFs were added to complete systems on the basis of their strong similarity to a partner in the system.

Some ABC systems were found to be located in the genome islands which were previously identified in *B. pseudomallei *K96243 [5]. The orthologous ABC system component proteins of *B. pseudomallei *K96243 and *B. mallei *ATCC 23344 were identified as proteins with > 90% identity when the ABC proteins of *B. mallei *ATCC 23344 were compared by BLASTP [38].

## Authors' contributions

DH participated in the conception, design and performance of bioinformatics studies and drafted the manuscript. ED contributed significantly to the design and performance of the bioinformatics studies. RT and KB participated in the conception of the study and critically revised the manuscript for important intellectual content. HA participated in the conception and design of the study and critically revised the manuscript for important intellectual content. This work was supported by Dstl. All authors have read and approved the final manuscript

## Supplementary Material

Additional file 1 Table 3 Complete ABC system inventories of *Bukholderia pseudomallei *K96243 and *Burkholderia mallei *ATCC 23344. The data provided represents the complete ABC system inventories for *Bukholderia pseudomallei *K96243 and *Burkholderia mallei *ATCC 23344. Click here for file

## Figures and Tables

**Table 1:** ABC systems present in *B. pseudomallei *and absent in *B. mallei*

Family/Subfamily^a^	Allocrite/Function	*B. pseudomallei *K96243	*B. mallei *ATCC 23344	No. of similar systems functioning
DPL/HLY	Haemolysin	BPSL1660/1664/1665	System absent	2
DPL/HMT	Fe/S cluster precursor	BPSL1797	System absent	2
DPL/SID	Pyochelin	BPSS0589^b^/0590	System absent	0
DRI/YHIH	Unknown	BPSS1937/1938/1939	Pseudogene	0
HAA	Branched-chain amino acids	BPSS0575/0576/0577/0578/0579/0802	Components absent	5
ISB	Unknown	BPSL2369/2370/2371	Pseudogene	0
ISVH	Fe(III)-pyochelin	BPSS0591/BPSL1781/1783/1784	Component absent	0
ISVH	Haemin	BPSL2721/2722/2723/2724	Pseudogene	1
MOI	Iron (III)	BPSS0702/0703/0704	Pseudogene	2
MOI	Polyamines	BPSL1649/1650/1651/1652 (on genomic island)	System absent	2
MOI	Polyamines	BPSS0075/0076/0077	System absent	2
MOI	Polyamines	BPSS0464/0465/0466/0467	System absent	2
MOS	Monosaccharide	BPSS0140/0141/0142	System absent	2
MOS	Monosaccharide	BPSS1030/1031/1032/1033	Components absent	2
MOS	Monosaccharide	BPSS2069/2070/2071 (on genomic island)	System absent	2
MOS	Ribose	BPSL1832/1833/1834	System absent	1
MOS	Ribose	BPSS0255/0256/0257	Pseudogene	1
o228	Unknown	BPSS0623/0624/0625	System absent	4
OSP	Oligosaccharide or polyol	BPSS2082/2083/2084/2085 (on genomic island)	System absent	1
OTCN	Aliphatic sulphonates	BPSL1822/1823/1824	System absent	2
OTCN	Nitrate	BPSL0712/0713	System absent	1
OTCN	Taurine	BPSS1572/1573/1574	Pseudogene	0
PAO	Polar amino acid	BPSL1807/1808/1809	System absent	0
PHN	Alkylphosphonate	BPSL2848/2849/2850	Pseudogene	0

^a ^Families and subfamilies as described at the ABSCISSE database [34]; ^b ^[27]

**Table 2:** Iron acquisition-associated ABC systems of *B. pseudomallei *and *B. mallei*.

Family/Subfamily^a^	Allocrite/Function	Type	*B. pseudomallei *K96243	*B. mallei *ATCC 23344	Comments
DPL/LAE	Ornibactin	IM-ABC^b^	BPSL1779	BMA1183	

ISVH	Hemin	ABC	BPSS0240	BMAA1830	
ISVH	Hemin	IM^c^	BPSS0241	BMAA1829	
ISVH	Hemin	BP^d^	BPSS0242	BMAA1828	
ISVH	Hemin	OMR^e^	BPSS0244	BMAA1826	
ISVH	Hemin	OMR	BPSS1742	BMAA0427	
ISVH	Malleobactin	OMR	BPSL1775^f^	BMA1178	
ISVH	Siderophore	OMR	BPSS1029	BMAA1180	
ISVH	Siderophore	OMR	BPSS1204		No BM^g ^ortholog
ISVH	Siderophore	OMR	BPSS1850	BMAA0251	

MOI	Iron (III)	ABC	BPSL1276	BMA1779	
MOI	Iron (III)	IM2^h^	BPSL1277	BMA1778	
MOI	Iron (III)	BP	BPSL1278	BMA1777	

MOI	Iron (III)	ABC	BPSL1300	BMA3076	
MOI	Iron (III)	IM	BPSL1301	BMA3075	
MOI	Iron (III)	IM	BPSL1302	BMA3072	
MOI	Iron (III)	BP	BPSL1303	BMA3071	

^a ^Families and subfamilies as described at the ABSCISSE database [34]; ^b ^inner membrane protein fused to N-terminal of ABC;^c ^inner membrane protein; ^d ^binding protein; ^e ^outer membrane receptor; ^f ^[27]; ^g ^*B. mallei *ATCC 23344; ^h ^2 fused inner membrane proteins.
