# Supplementary material for: ATP-binding cassette systems in Burkholderia pseudomallei and Burkholderia mallei
Source: BMC Genomics. 2007 Mar 28;8:83. doi: 10.1186/1471-2164-8-83 (PMC1853089; doi:10.1186/1471-2164-8-83)
Supplement: Additional file 1 — Table 3 Complete ABC system inventories of Bukholderia pseudomallei K96243 and Burkholderia mallei ATCC 23344. The data provided represents the complete ABC system inventories for Bukholderia pseudomallei K96243 and Burkholderia mallei ATCC 23344. [file 1471-2164-8-83-S1.doc]

| 1 | ART | ARE | Unknown | MLS antibiotic resistance | ABC2 | BPSL0375 | BMA0092 |  |
| --- | --- | --- | --- | --- | --- | --- | --- | --- |
| 2 | ART | REG | Unknown | Unknown | ABC2 | BPSL1068 | BMA2207 |  |
| 3 | ART | REG | Unknown | Unknown | ABC2 | BPSL1548 | BMA1308 |  |
| 4 | ART | REG | Unknown | Unknown | ABC2 | BPSL2176 | BMA1572 |  |
| 5 | ART | REG | Unknown | Unknown | ABC2 | BPSL3067 | BMAA0510 | On ≠ chromososmes |
| 6 | CLS |  | Capsular polysaccharide |  | ABC | BPSL2804 (*wzt2*) | BMA2304 |  |
|  | CLS |  | Capsular polysaccharide |  | IM | BPSL2805 (*wzm2*) | BMA2305 |  |
|  | CLS |  | Capsular polysaccharide |  | MPA | BPSL2806 (*wcbD*) |  |  |
|  | CLS |  | Capsular polysaccharide |  | OMA | BPSL2807 (*wcbC*) | BMA2307 |  |
| 7 | CLS |  | Lipopolysaccharide |  | ABC | BPSL2681 (*wzt*) | BMA1985 |  |
|  | CLS |  | Lipopolysaccharide |  | IM | BPSL2682 (*wzm*) | BMA1986 |  |
| 8 | DLM |  | D L Methionine |  | BP | BPSL2501 | BMA0412 |  |
|  | DLM |  | D L Methionine |  | IM | BPSL2502 | BMA0413 |  |
|  | DLM |  | D L Methionine |  | ABC | BPSL2503 | BMA0414 |  |
|  | DLM |  | D L Methionine |  | BP | BPSL0466 | BMA3183 |  |
|  | DLM |  | D L Methionine |  | BP | BPSS0266 | BMAA1501 |  |
| 9 | DLM |  | D L Methionine |  | BP | BPSS0313 | BMAA1441 |  |
|  | DLM |  | D L Methionine |  | ABC | BPSS0315 | BMAA1439 |  |
|  | DLM |  | D L Methionine |  | IM | BPSS0316 | BMAA1438 |  |
| 10 | DPL | HLY | Hemolysin |  | OMP | BPSL1660 |  |  |
|  | DPL | HLY | Hemolysin |  | IM-ABC | BPSL1664 |  |  |
|  | DPL | HLY | Hemolysin |  | MFP | BPSL1665 |  |  |
| 11 | DPL | HLY | Hemolysin |  | MFP | BPSL3092 | BMA2820 |  |
|  | DPL | HLY | Hemolysin |  | IM-ABC | BPSL3093 | BMA2821 |  |
|  | DPL | HLY | Hemolysin |  | OMP | BPSL3094 | BMA2822 |  |
| 12 | DPL | HLY | Hemolysin |  | MFP | BPSS1283 | BMAA1002 |  |
|  | DPL | HLY | Hemolysin |  | IM-ABC | BPSS1285 | BMAA1000 |  |
|  | DPL | HLY | Hemolysin |  | OMP | BPSS1287 | BMAA0997 |  |
| 13 | DPL | HMT | Fe/S cluster precursors |  | IM-ABC | BPSL0655 | BMA0204 |  |
| 14 | DPL | HMT | Fe/S cluster precursors |  | IM-ABC | BPSL1797 |  | No BM homologue |
| 15 | DPL | HMT | Fe/S cluster precursors |  | IM-ABC | BPSS2259 | BMAA1994 | BM C-term longer & ≠ |
| 16 | DPL | HMT | Unknown |  | IM-ABC | BPSL2409 | BMA0567 | BP N-term ≠ |
| 17 | DPL | HMT | Unknown |  | IM-ABC | BPSS1862 | BMAA0233 |  |
| 18 | DPL | LAE | Malleobactin |  | IM-ABC | BPSL1779 (*mbaE*) | BMA1183 | BP N-term ≠ |
| 19 | DPL | LIP | Lipid A |  | ENZ | BPSL0878 | BMA1912 |  |
|  | DPL | LIP | Lipid A |  | IM-ABC | BPSL1118 | BMA2273 |  |
| 20 | DPL | SID | Pyochelin? |  | IM-ABC | BPSS0589 |  |  |
|  | DPL | SID | Pyochelin? |  | IM-ABC | BPSS0590 |  |  |
| 21 | DRA | DRR | Unknown | Drug resistance | IM | BPSL0478 | BMA3151 |  |
|  | DRA | DRR | Unknown | Drug resistance | ABC | BPSL0479 | BMA3152 |  |
| 22 | DRA | DRR | Unknown | Drug resistance | IM | BPSL3143 | BMA2718 |  |
|  | DRA | DRR | Unknown | Drug resistance | ABC | BPSL3144 | BMA2719 |  |
| 23 | DRA | NOD | Unknown | Nodulation? | ABC | BPSL1844 | BMA1218 |  |
|  | DRA | NOD | Unknown | Nodulation? | IM | BPSL1845 | BMA1219 |  |
| 24 | DRI | DRB | Unknown | Drug resistance | ABC | BPSL1609 | BMA0997 |  |
|  | DRI | DRB | Unknown | Drug resistance | IM | BPSL1610 | BMA0998 |  |
| 25 | DRI | NOS | Unknown | Nitric oxide reduction | IM | BPSL1604 | BMA0992 |  |
|  | DRI | NOS | Unknown | Nitric oxide reduction | ABC | BPSL1605 | BMA0993 |  |
|  | DRI | NOS | Unknown | Nitric oxide reduction | SS | BPSL1606 | BMA0994 |  |
| 26 | DRI | YHIH | Unknown | Drug resistance | MFP | BPSS1937 |  | BM pseudogene |
|  | DRI | YHIH | Unknown | Drug resistance | ABC2-IM | BPSS1938 | BMAA0139 |  |
|  | DRI | YHIH | Unknown | Drug resistance | IM | BPSS1939 | BMAA0138 |  |
| 27 | DPL | FAE | Unknown | Toxin export | IM-ABC | BPSL3155 | BMA2731 |  |
| 28 | HAA |  | Amides and urea |  | BP | BPSL2651 | BMA2176 |  |
|  | HAA |  | Amides and urea |  | IM | BPSL2652 | BMA2177 |  |
|  | HAA |  | Amides and urea |  | IM | BPSL2653 | BMA2178 |  |
|  | HAA |  | Amides and urea |  | ABC | BPSL2654 | BMA2179 |  |
|  | HAA |  | Amides and urea |  | ABC | BPSL2655 | BMA2180 |  |
| 29 | HAA |  | Branched-chain amino acids |  | ABC | BPSL0046 | BMA2669 |  |
|  | HAA |  | Branched-chain amino acids |  | ABC | BPSL0047 | BMA2668 |  |
|  | HAA |  | Branched-chain amino acids |  | IM | BPSL0048 | BMA2667 |  |
|  | HAA |  | Branched-chain amino acids |  | IM | BPSL0049 | BMA2665 |  |
|  | HAA |  | Branched-chain amino acids |  | BP | BPSL0050 | BMA2666 |  |
| 30 | HAA |  | Branched-chain amino acids |  | IM | BPSL0920 | BMA1973 |  |
|  | HAA |  | Branched-chain amino acids |  | IM | BPSL0921 | BMA1972 |  |
|  | HAA |  | Branched-chain amino acids |  | ABC | BPSL0922 | BMA1971 |  |
|  | HAA |  | Branched-chain amino acids |  | ABC | BPSL0923 | BMA1970 |  |
|  | HAA |  | Branched-chain amino acids |  | BP | BPSL1205 | BMA1836 |  |
|  | HAA |  | Branched-chain amino acids |  | BP | BPSL1751 | BMA1153 |  |
| 31 | HAA |  | Branched-chain amino acids |  | ABC | BPSL1998 | BMA0907 |  |
|  | HAA |  | Branched-chain amino acids |  | IM-ABC | BPSL1999 | BMA0908 |  |
|  | HAA |  | Branched-chain amino acids |  | IM | BPSL2000 | BMA0909 |  |
|  | HAA |  | Branched-chain amino acids |  | BP | BPSL2404 | BMA0572 |  |
|  | HAA |  | Branched-chain amino acids |  | BP | BPSL3284 | BMA2901 |  |
|  | HAA |  | Branched-chain amino acids |  | BP | BPSL3388 | BMA2966 |  |
| 32 | HAA |  | Branched-chain amino acids |  | ABC | BPSL3409 | BMA2943 |  |
|  | HAA |  | Branched-chain amino acids |  | IM-ABC | BPSL3410 | BMA2942 |  |
|  | HAA |  | Branched-chain amino acids |  | IM | BPSL3411 | BMA2941 |  |
|  | HAA |  | Branched-chain amino acids |  | BP | BPSL3412 | BMA2940 |  |
| 33 | HAA |  | Branched-chain amino acids |  | IM | BPSL3414 | BMA2938 |  |
|  | HAA |  | Branched-chain amino acids |  | IM | BPSL3415 | BMA2937 |  |
|  | HAA |  | Branched-chain amino acids |  | BP | BPSL3416 | BMA2936 |  |
|  | HAA |  | Branched-chain amino acids |  | ABC | BPSL3417 | BMA2935 | BM N term truncated |
|  | HAA |  | Branched-chain amino acids |  | ABC | BPSL3418 | BMA2934 |  |
| 34 | HAA |  | Branched-chain amino acids |  | BP | BPSS0575 |  |  |
|  | HAA |  | Branched-chain amino acids |  | IM | BPSS0576 |  |  |
|  | HAA |  | Branched-chain amino acids |  | IM | BPSS0577 |  |  |
|  | HAA |  | Branched-chain amino acids |  | ABC | BPSS0578 |  |  |
|  | HAA |  | Branched-chain amino acids |  | ABC | BPSS0579 |  |  |
|  | HAA |  | Branched-chain amino acids |  | BP | BPSS0802 | BMAA0660 |  |
| 35 | ISB |  | Unknown | Fe/S centre biogenesis | CYT | BPSL2369 |  | BM pseudogene |
|  | ISB |  | Unknown | Fe/S centre biogenesis | ABC | BPSL2370 | BMA0612 |  |
|  | ISB |  | Unknown | Fe/S centre biogenesis | CYT | BPSL2371 | BMA0611 |  |
| 36 | ISVH |  | Fe(III)-pyochelin |  | OMR | BPSS0591 |  | No BM homologue |
|  | ISVH |  | Ferric citrate or Ornibactin |  | BP | BPSL1781 | BMA1185 |  |
|  | ISVH |  | Ferric citrate or Ornibactin |  | IM | BPSL1783 | BMA1187 |  |
|  | ISVH |  | Ferric citrate or Ornibactin |  | ABC | BPSL1784 | BMA1188 |  |
| 37 | ISVH |  | Hemin |  | IM | BPSL2721 | BMA2033 |  |
|  | ISVH |  | Hemin |  | BP | BPSL2722 |  | BM pseudogene |
|  | ISVH |  | Hemin |  | ABC | BPSL2723 | BMA2035 |  |
|  | ISVH |  | Hemin |  | OMR | BPSL2724 | BMA2036 |  |
| 38 | ISVH |  | Hemin |  | ABC | BPSS0240 | BMAA1830 |  |
|  | ISVH |  | Hemin |  | IM | BPSS0241 | BMAA1829 |  |
|  | ISVH |  | Hemin |  | BP | BPSS0242 | BMAA1828 |  |
|  | ISVH |  | Hemin |  | OMR | BPSS0244 | BMAA1826 |  |
|  | ISVH |  | Hemin |  | OMR | BPSS1742 | BMAA0427 | Annotated copper |
|  | ISVH |  | Malleobactin |  | OMR | BPSL1775 (*fmtA*) | BMA1178 |  |
|  | ISVH |  | Siderophore |  | OMR | BPSS1029 | BMAA1180 |  |
|  | ISVH |  | Siderophore |  | OMR | BPSS1204 |  | No BM homologue |
|  | ISVH |  | Siderophore |  | OMR | BPSS1850 | BMAA0251 |  |
| 39 | ISVH |  | Vitamin B-12 |  | OMR | BPSL0976 | BMA0685 |  |
|  | ISVH |  | Vitamin B-12 |  | IM | BPSL0977 | BMA0686 |  |
|  | ISVH |  | Vitamin B-12 |  | ABC | BPSL0978 | BMA0687 |  |
|  | ISVH |  | Vitamin B-12 |  | BP | BPSL0983 | BMA0693 |  |
|  | ISVH |  | Vitamin B-12 |  | OMR | BPSL2553 | BMA0477 |  |
| 40 | MET |  | Metallic cation |  | IM | BPSL0822 | BMA0326 |  |
|  | MET |  | Metallic cation |  | ABC | BPSL0823 | BMA0327 |  |
|  | MET |  | Metallic cation |  | BP | BPSL0824 | BMA0328 |  |
| 41 | MKL |  | Unknown |  | IM | BPSL0401 | BMA0066 |  |
|  | MKL |  | Unknown |  | ABC | BPSL0402 | BMA0065 |  |
|  | MKL |  | Unknown |  | SS | BPSL0403 | BMA0064 |  |
| 42 | MKL |  | Unknown |  | SS | BPSL3146 | BMA2722 |  |
|  | MKL |  | Unknown |  | SS | BPSL3148 | BMA2724 |  |
|  | MKL |  | Unknown |  | IM | BPSL3149 | BMA2726 |  |
|  | MKL |  | Unknown |  | ABC | BPSL3150 | BMA2725 |  |
|  | MKL |  | Unknown |  | SS | BPSS2316 | BMAA2076 |  |
| 43 | MOI |  | 2-aminoethylphosphonate |  | BP | BPSS0344 | BMAA0855 |  |
|  | MOI |  | 2-aminoethylphosphonate |  | ABC | BPSS0345 | BMAA0856 |  |
|  | MOI |  | 2-aminoethylphosphonate |  | IM | BPSS0346 | BMAA0857 |  |
|  | MOI |  | 2-aminoethylphosphonate |  | IM | BPSS0347 | BMAA0858 | BM in-frame insertion |
| 44 | MOI |  | Iron (III) |  | ABC | BPSL1276 | BMA1779 |  |
|  | MOI |  | Iron (III) |  | IM2 | BPSL1277 | BMA1778 |  |
|  | MOI |  | Iron (III) |  | BP | BPSL1278 | BMA1777 |  |
| 45 | MOI |  | Iron (III) |  | ABC | BPSL1300 | BMA3076 |  |
|  | MOI |  | Iron (III) |  | IM | BPSL1301 | BMA3075 | BM C-term ≠ |
|  | MOI |  | Iron (III) |  | IM | BPSL1302 | BMA3072 |  |
|  | MOI |  | Iron (III) |  | BP | BPSL1303 | BMA3071 |  |
| 46 | MOI |  | Iron (III) |  | ABC | BPSS0702 | BMAA1130 |  |
|  | MOI |  | Iron (III) |  | IM | BPSS0703 |  | BM pseudogene |
|  | MOI |  | Iron (III) |  | BP | BPSS0704 | BMAA1128 |  |
| 47 | MOI |  | Mineral or organic ion or Iron |  | IM | BPSS0247 | BMAA1822 |  |
|  | MOI |  | Mineral or organic ion or Iron |  | ABC | BPSS0248 | BMAA1821 |  |
|  | MOI |  | Mineral or organic ion or Iron |  | BP | BPSS0249 | BMAA1820 |  |
|  | MOI |  | Mineral or organic ion or Iron |  | IM | BPSS0250 | BMAA1819 |  |
| 48 | MOI |  | Molybdate |  | BP | BPSL2726 | BMA2039 |  |
|  | MOI |  | Molybdate |  | BP | BPSS1786 | BMAA0299 |  |
|  | MOI |  | Molybdate |  | IM | BPSS1787 | BMAA0298 |  |
|  | MOI |  | Molybdate |  | ABC | BPSS1788 | BMAA0297 |  |
| 49 | MOI |  | Phosphate |  | BP | BPSL1359 | BMA0780 |  |
|  | MOI |  | Phosphate |  | IM | BPSL1360 | BMA0781 |  |
|  | MOI |  | Phosphate |  | IM | BPSL1361 | BMA0782 |  |
|  | MOI |  | Phosphate |  | ABC | BPSL1362 | BMA0783 |  |
| 50 | MOI |  | Polyamines |  | BP | BPSL1649 |  |  |
|  | MOI |  | Polyamines |  | IM | BPSL1650 |  | Present on genomic |
|  | MOI |  | Polyamines |  | IM | BPSL1651 |  | island 8 in BP |
|  | MOI |  | Polyamines |  | ABC | BPSL1652 |  |  |
| 51 | MOI |  | Polyamines |  | IM | BPSS0075 |  |  |
|  | MOI |  | Polyamines |  | IM | BPSS0076 |  |  |
|  | MOI |  | Polyamines |  | BP | BPSS0077 |  |  |
| 52 | MOI |  | Polyamines |  | IM | BPSS0464 |  |  |
|  | MOI |  | Polyamines |  | IM | BPSS0465 |  |  |
|  | MOI |  | Polyamines |  | ABC | BPSS0466 |  |  |
|  | MOI |  | Polyamines |  | BP | BPSS0467 |  |  |
| 53 | MOI |  | Polyamines |  | IM | BPSS1735 |  | BM pseudogene |
|  | MOI |  | Polyamines |  | IM | BPSS1736 | BMAA0433 |  |
|  | MOI |  | Polyamines |  | ABC | BPSS1737 | BMAA0432 |  |
|  | MOI |  | Polyamines |  | BP | BPSS1738 | BMAA0431 |  |
| 54 | MOI |  | Polyamines |  | BP | BPSL1555 | BMA1301 |  |
|  | MOI |  | Polyamines |  | ABC | BPSL1556 | BMA1300 |  |
|  | MOI |  | Polyamines |  | IM | BPSL1557 | BMA1299 |  |
|  | MOI |  | Polyamines |  | IM | BPSL1558 | BMA1298 |  |
|  | MOI |  | Polyamines |  | BP | BPSL0108 | BMA0123 |  |
| 55 | MOI |  | Thiosulfate/sulfate |  | BP | BPSL0352 |  | No BM homologue |
|  | MOI |  | Thiosulfate/sulfate |  | ABC | BPSL1836 | BMA1206 |  |
|  | MOI |  | Thiosulfate/sulfate |  | IM | BPSL1837 | BMA1207 |  |
|  | MOI |  | Thiosulfate/sulfate |  | IM | BPSL1838 | BMA1208 |  |
|  | MOI |  | Thiosulfate/sulfate |  | BP | BPSL1839 | BMA1209 |  |
| 56 | MOS |  | Arabinose |  | IM | BPSL2966 (*araH*) | BMA2484 |  |
|  | MOS |  | Arabinose |  | ABC2 | BPSL2967 (*araG*) | BMA2485 |  |
|  | MOS |  | Arabinose |  | BP | BPSL2968 (*araF*) | BMA2486 |  |
|  | MOS |  | Arabinose |  | BP | BPSS1220 | BMAA1057 |  |
| 57 | MOS |  | Monosaccharide |  | BP | BPSL1991 | BMA0916 |  |
|  | MOS |  | Monosaccharide |  | IM | BPSL1992 | BMA0915 |  |
|  | MOS |  | Monosaccharide |  | ABC | BPSL1993 | BMA0914 |  |
| 58 | MOS |  | Monosaccharide |  | BP | BPSS0140 |  |  |
|  | MOS |  | Monosaccharide |  | IM | BPSS0141 |  |  |
|  | MOS |  | Monosaccharide |  | ABC2 | BPSS0142 |  |  |
| 59 | MOS |  | Monosaccharide |  | BP | BPSS0786 | BMAA0637 |  |
|  | MOS |  | Monosaccharide |  | ABC2 | BPSS0787 | BMAA0638 |  |
|  | MOS |  | Monosaccharide |  | IM | BPSS0788 | BMAA0639 |  |
| 60 | MOS |  | Monosaccharide |  | IM | BPSS1030 | BMAA1179 |  |
|  | MOS |  | Monosaccharide |  | IM | BPSS1031 |  |  |
|  | MOS |  | Monosaccharide |  | ABC2 | BPSS1032 |  |  |
|  | MOS |  | Monosaccharide |  | BP | BPSS1033 |  |  |
| 61 | MOS |  | Monosaccharide |  | ABC | BPSS2069 |  | Present on genomic |
|  | MOS |  | Monosaccharide |  | IM | BPSS2070 |  | island 16 in BP |
|  | MOS |  | Monosaccharide |  | BP | BPSS2071 |  |  |
| 62 | MOS |  | Ribose |  | IM | BPSL1791 | BMA1196 |  |
|  | MOS |  | Ribose |  | ABC2 |  | BMA1197 |  |
|  | MOS |  | Ribose |  | BP | BPSL1793 | BMA1198 |  |
| 63 | MOS |  | Ribose |  | IM | BPSL1832 |  |  |
|  | MOS |  | Ribose |  | ABC2 | BPSL1833 |  |  |
|  | MOS |  | Ribose |  | BP | BPSL1834 |  |  |
| 64 | MOS |  | Ribose |  | ABC | BPSS0255 | BMAA1814 |  |
|  | MOS |  | Ribose |  | IM | BPSS0256 | BMAA1813 | BM 31AA deletion |
|  | MOS |  | Ribose |  | BP | BPSS0257 | BMAA1812 |  |
| 65 | NEW1 |  | Unknown | Unknown | BP | BPSL0467 | BMA3140 |  |
|  | NEW1 |  | Unknown | Unknown | IM | BPSL0468 | BMA3141 |  |
|  | NEW1 |  | Unknown | Unknown | ABC | BPSL0469 | BMA3142 |  |
| 66 | NO | PHNK | Unknown | Unknown | ABC | BPSL2853 | BMA2404 |  |
| 67 | NO | PHNL | Unknown | Unknown | ABC | BPSL2852 | BMA2405 | BM N term truncated |
| 68 | NO |  | Unknown | Unknown | IM | BPSL1545 | BMA1310 |  |
|  | NO |  | Unknown | Unknown | ABC | BPSL1546 | BMA1311 |  |
| 69 | o228 |  | Unknown | Unknown | ABC | BPSL1412 | BMA1450 |  |
| 70 | o228 |  | Unknown | Lipoprotein release | ABC | BPSL2276 | BMA1695 |  |
|  | o228 |  | Unknown | Lipoprotein release | IM | BPSL2277 | BMA1696 |  |
| 71 | o228 |  | Unknown | Drug resistance | OMP | BPSS0623 |  |  |
|  | o228 |  | Unknown | Drug resistance | ABC-IM | BPSS0624 |  |  |
|  | o228 |  | Unknown | Drug resistance | MFP | BPSS0625 |  |  |
| 72 | o228 |  | Unknown | Drug resistance | MFP | BPSS1930 | BMAA0148 |  |
|  | o228 |  | Unknown | Drug resistance | ABC | BPSS1931 | BMAA0147 |  |
|  | o228 |  | Unknown | Drug resistance | IM | BPSS1932 | BMAA0146 |  |
| 73 | o228 |  | Unknown | Unknown | IM | BPSS2324 | BMAA2085 |  |
|  | o228 |  | Unknown | Unknown | ABC | BPSS2325 | BMAA2086 |  |
| 74 | OPN |  | Dipeptides |  | BP | BPSL0249 | BMA3301 |  |
|  | OPN |  | Dipeptides |  | IM | BPSL0250 | BMA3302 |  |
|  | OPN |  | Dipeptides |  | IM | BPSL0251 |  | BM pseudogene |
|  | OPN |  | Dipeptides |  | ABC | BPSL0252 | BMA3304 |  |
|  | OPN |  | Dipeptides |  | ABC | BPSL0253 | BMA3305 |  |
|  | OPN |  | Oligopeptides |  | BP | BPSL0376 | BMA0091 |  |
| 75 | OPN |  | Oligopeptides |  | ABC2 | BPSL2200 | BMA1604 |  |
|  | OPN |  | Oligopeptides |  | IM | BPSL2201 | BMA1605 |  |
|  | OPN |  | Oligopeptides |  | IM | BPSL2202 | BMA1606 |  |
|  | OPN |  | Oligopeptides |  | BP | BPSL2203 | BMA1607 |  |
| 76 | OPN |  | Oligopeptides |  | ABC | BPSS0950 | BMAA1278 |  |
|  | OPN |  | Oligopeptides |  | ABC | BPSS0951 | BMAA1277 |  |
|  | OPN |  | Oligopeptides |  | IM | BPSS0952 | BMAA1276 |  |
|  | OPN |  | Oligopeptides |  | IM | BPSS0953 | BMAA1275 |  |
|  | OPN |  | Oligopeptides |  | BP | BPSS0954 | BMAA1274 |  |
| 77 | OPN |  | Oligopeptides |  | IM | BPSS1304 | BMAA0977 |  |
|  | OPN |  | Oligopeptides |  | IM | BPSS1305 | BMAA0976 |  |
|  | OPN |  | Oligopeptides |  | BP | BPSS1306 | BMAA0975 |  |
|  | OPN |  | Oligopeptides |  | ABC2 | BPSS1307 | BMAA0974 |  |
| 78 | OPN |  | Oligopeptides |  | ABC | BPSS2137 |  | No BM homologue |
|  | OPN |  | Oligopeptides |  | ABC | BPSS2138 | BMAA0354 |  |
|  | OPN |  | Oligopeptides |  | IM | BPSS2139 | BMAA0353 |  |
|  | OPN |  | Oligopeptides |  | IM | BPSS2140 | BMAA0352 |  |
|  | OPN |  | Oligopeptides |  | BP | BPSS2141 | BMAA0351 |  |
|  | OPN |  | Oligopeptides |  | BP | BPSS0964 | BMAA1261 |  |
| 79 | OSP |  | Glucose |  | ABC | BPSL2608 | BMA2126 |  |
|  | OSP |  | Glucose |  | IM | BPSL2609 | BMA2127 |  |
|  | OSP |  | Glucose |  | IM | BPSL2610 | BMA2128 |  |
|  | OSP |  | Glucose |  | BP | BPSL2611 | BMA2129 |  |
| 80 | OSP |  | Glycerol-3-phosphate |  | ABC | BPSL3163 | BMA2741 |  |
|  | OSP |  | Glycerol-3-phosphate |  | IM | BPSL3164 | BMA2742 |  |
|  | OSP |  | Glycerol-3-phosphate |  | IM | BPSL3165 | BMA2743 |  |
|  | OSP |  | Glycerol-3-phosphate |  | BP | BPSL3166 | BMA2744 |  |
| 81 | OSP |  | Mannitol/Arabitol/Glucitol |  | BP | BPSL0829 | BMA0333 |  |
|  | OSP |  | Mannitol/Arabitol/Glucitol |  | IM | BPSL0830 | BMA0334 |  |
|  | OSP |  | Mannitol/Arabitol/Glucitol |  | IM | BPSL0831 | BMA0335 |  |
|  | OSP |  | Mannitol/Arabitol/Glucitol |  | ABC | BPSL0833 | BMA0337 |  |
| 82 | OSP |  | Oligosaccharide or polyol |  | BP | BPSS1232 | BMAA1073 |  |
|  | OSP |  | Oligosaccharide or polyol |  | IM | BPSS1233 | BMAA1074 |  |
|  | OSP |  | Oligosaccharide or polyol |  | IM | BPSS1234 | BMAA1075 |  |
|  | OSP |  | Oligosaccharide or polyol |  | ABC | BPSS1236 | BMAA1077 |  |
| 83 | OSP |  | Oligosaccharide or polyol |  | IM | BPSS2082 |  |  |
|  | OSP |  | Oligosaccharide or polyol |  | IM | BPSS2083 |  | Present on genomic |
|  | OSP |  | Oligosaccharide or polyol |  | BP | BPSS2084 |  | island 16 in BP |
|  | OSP |  | Oligosaccharide or polyol |  | ABC | BPSS2085 |  |  |
| 84 | OTCN |  | Aliphatic sulfonates |  | BP | BPSL0036 | BMA2680 |  |
|  | OTCN |  | Aliphatic sulfonates |  | ABC | BPSL0037 | BMA2679 |  |
|  | OTCN |  | Aliphatic sulfonates |  | IM | BPSL0038 | BMA2678 |  |
| 85 | OTCN |  | Aliphatic sulfonates |  | IM | BPSL1822 |  |  |
|  | OTCN |  | Aliphatic sulfonates |  | ABC | BPSL1823 |  |  |
|  | OTCN |  | Aliphatic sulfonates |  | BP | BPSL1824 |  |  |
| 86 | OTCN |  | Aliphatic sulfonates |  | ABC | BPSL1856 | BMA1237 |  |
|  | OTCN |  | Aliphatic sulfonates |  | IM | BPSL1857 | BMA1238 |  |
|  | OTCN |  | Aliphatic sulfonates |  | BP | BPSL1252 |  | No BM homologue |
|  | OTCN |  | Aliphatic sulfonates |  | BP | BPSS0928 | BMAA1303 |  |
| 87 | OTCN |  | Choline |  | BP | BPSL1737 | BMA1138 |  |
|  | OTCN |  | Choline |  | IM | BPSL1738 | BMA1139 |  |
|  | OTCN |  | Choline |  | ABC | BPSL1739 | BMA1140 |  |
|  | OTCN |  | Choline |  | IM | BPSL1740 | BMA1141 |  |
| 88 | OTCN |  | Glycine-Betaine Proline |  | BP | BPSS0556 | BMAA0480 |  |
|  | OTCN |  | Glycine-Betaine Proline |  | BP | BPSS0561 | BMAA0485 |  |
|  | OTCN |  | Glycine-Betaine Proline |  | BP | BPSS0566 | BMAA0490 | lipoprotein |
|  | OTCN |  | Glycine-Betaine Proline |  | BP | BPSS1423 | BMAA0567 |  |
|  | OTCN |  | Glycine-Betaine Proline |  | IM | BPSS1425 | BMAA0565 |  |
|  | OTCN |  | Glycine-Betaine Proline |  | ABC | BPSS1426 | BMAA0564 |  |
| 89 | OTCN |  | Nitrate? |  | ABC | BPSL0712 |  |  |
|  | OTCN |  | Nitrate? |  | IM2 | BPSL0713 |  |  |
| 90 | OTCN |  | Nitrate? |  | IM2 | BPSL1039 | BMA0754 |  |
|  | OTCN |  | Nitrate? |  | ABC | BPSL1040 | BMA0755 |  |
| 91 | OTCN |  | Taurine |  | IM | BPSS1572 | BMAA1580 |  |
|  | OTCN |  | Taurine |  | ABC | BPSS1573 | BMAA1581 |  |
|  | OTCN |  | Taurine |  | BP | BPSS1574 | BMAA1582 | BM N term truncated |
| 92 | PAO |  | Cystine and diaminopimelic acid |  | BP | BPSL1867 | BMA1254 |  |
|  | PAO |  | Cystine and diaminopimelic acid |  | IM | BPSL1868 | BMA1255 |  |
| 93 | PAO |  | Cystine and diaminopimelic acid |  | BP | BPSL2615 | BMA2134 |  |
|  | PAO |  | Cystine and diaminopimelic acid |  | IM | BPSL2616 | BMA2135 |  |
|  | PAO |  | Cystine and diaminopimelic acid |  | ABC | BPSL2617 | BMA2136 |  |
| 94 | PAO |  | Cystine and diaminopimelic acid |  | BP | BPSS1670 | BMAA1686 |  |
|  | PAO |  | Cystine and diaminopimelic acid |  | IM-ABC | BPSS1671 | BMAA1688 |  |
|  | PAO |  | Cystine and diaminopimelic acid |  | BP | BPSS1674 | BMAA1691 |  |
| 95 | PAO |  | Glutamate |  | ABC | BPSL2921 | BMA2434 |  |
|  | PAO |  | Glutamate |  | IM | BPSL2922 | BMA2435 |  |
|  | PAO |  | Glutamate |  | IM | BPSL2923 | BMA2436 |  |
|  | PAO |  | Glutamate |  | BP | BPSL2924 | BMA2437 | BM C term trunc ISBma1 |
|  | PAO |  | Glutamate |  | BP | BPSS0153 | BMAA1934 |  |
| 96 | PAO |  | Glutamine |  | ABC | BPSS2335 | BMAA2096 |  |
|  | PAO |  | Glutamine |  | IM | BPSS2336 | BMAA2097 |  |
|  | PAO |  | Glutamine |  | BP | BPSS2337 | BMAA2098 |  |
| 97 | PAO |  | Histidine |  | ABC | BPSL1030 | BMA0744 |  |
|  | PAO |  | Histidine |  | IM | BPSL1031 | BMA0745 |  |
|  | PAO |  | Histidine |  | IM | BPSL1032 | BMA0746 |  |
|  | PAO |  | Histidine |  | BP | BPSL1033 | BMA0747 |  |
| 98 | PAO |  | Histidine |  | BP | BPSL2383 | BMA0598 |  |
|  | PAO |  | Histidine |  | ABC | BPSL2392 | BMA0589 |  |
|  | PAO |  | Histidine |  | IM | BPSL2393 | BMA0588 |  |
|  | PAO |  | Histidine |  | IM | BPSL2394 |  | No BM homologue |
|  | PAO |  | Histidine |  | BP | BPSL3383 | BMA2972 |  |
| 99 | PAO |  | Histidine |  | BP | BPSS0269 | BMAA1496 |  |
|  | PAO |  | Histidine |  | IM | BPSS0981 | BMAA1240 |  |
|  | PAO |  | Histidine |  | IM | BPSS0982 | BMAA1238 |  |
|  | PAO |  | Histidine |  | ABC | BPSS0983 | BMAA1239 |  |
| 100 | PAO |  | Polar amino acid |  | IM | BPSL1807 |  |  |
|  | PAO |  | Polar amino acid |  | IM | BPSL1808 |  |  |
|  | PAO |  | Polar amino acid |  | BP | BPSL1809 |  |  |
| 101 | PHN |  | Alkylphosphonates |  | IM | BPSL2848 | BMA2407 |  |
|  | PHN |  | Alkylphosphonates |  | BP | BPSL2849 |  | BM pseudogene |
|  | PHN |  | Alkylphosphonates |  | ABC | BPSL2850 | BMA2409 |  |
| 102 | UVR |  | Unknown | DNA repair | ABC2 | BPSL0545 | BMA3095 |  |
| 103 | UVR |  | Unknown | DNA repair |  | BPSL2402 | BMA0552 |  |
| 104 | UVR |  | Unknown | DNA repair |  | BPSS0356 | BMAA0880 |  |
| 105 | UVR |  | Unknown | Unknown | ABC4 | BPSS0058 | BMAA0062 |  |
| 106 | YHBG |  | Unknown | Unknown | ABC | BPSL0534 | BMA3107 |  |
|  | YHBG |  | Unknown | Unknown | SS | BPSL0535 | BMA3105 |  |
|  | YHBG |  | Unknown | Unknown | SS | BPSL0536 | BMA3104 |  |
|  | YHBG |  | Unknown | Unknown | ENZ | BPSL0537 | BMA3103 |  |
|  |  |  |  |  |  |  |  |  |
